# Early-Stage Application of Agomir-137 Promotes Locomotor Recovery in a Mouse Model of Motor Cortex Injury

**DOI:** 10.3390/ijms242417156

**Published:** 2023-12-05

**Authors:** Xiao-Tian Liu, Zhao-Qian Teng

**Affiliations:** 1 State Key Laboratory of Stem Cell and Reproductive Biology, Institute of Zoology, Chinese Academy of Sciences, Beijing 100101, China; liuxt4096@163.com; 2 Savaid Medical School, University of Chinese Academy of Sciences, Beijing 100408, China; 3 Institute for Stem Cell and Regeneration, Chinese Academy of Sciences, Beijing 100101, China; 4 Beijing Institute for Stem Cell and Regenerative Medicine, Beijing 100101, China

**Keywords:** traumatic brain injury, miR-137, agomir, antagomir, locomotor recovery

## Abstract

Traumatic brain injury (TBI) is a significant risk factor for neurodegenerative disorders, and patients often experience varying degrees of motor impairment. MiR-137, a broadly conserved and brain-enriched miRNA, is a key regulator in neural development and in various neurological diseases. Following TBI, the expression of miR-137 is dramatically downregulated. However, whether miR-137 is a therapeutic target for TBI still remains unknown. Here, for the first time, we demonstrate that intranasal administration of miR-137 agomir (a mimic) in the early stage (0–7 days) of TBI effectively inhibits glial scar formation and improves neuronal survival, while early-stage administration of miR-137 antagomir (an inhibitor) deteriorates motor impairment. This study elucidates the therapeutic potential of miR-137 mimics in improving locomotor recovery following motor cortex injury.

## 1. Introduction

As one of the leading causes of death around world, traumatic brain injury (TBI) induces many types of neurodegenerative diseases accompanied by multiple disorders [1,2]. Locomotor deficiency induced by motor cortex injury is common among patients [3,4], and many negative mechanisms are involved in the pathological process, including excitotoxicity, reactive oxygen species (ROS) production, inflammation, ionic imbalance and apoptosis [5,6]. However, the molecular mechanisms underlying TBI-induced locomotor deficiency are still not well known, presenting obstacles for clinical treatment design and pharmaceutical interventions [7].

MicroRNAs are a type of non-coding RNA molecule that are formed by ~22 nucleotides and involved in post-transcriptional regulation of gene expression. It has been proved that microRNAs are essential for normal neuronal development, brain function and multiple neuronal diseases [8,9]. As for TBI, it is well known that many microRNAs are dysregulated and may serve as potential targets for the development of drugs and strategies for the clinical treatment of TBI patients [10,11,12,13]. 

The broadly conserved microRNA miR-137 is highly expressed in the brain [14,15,16]. miR-137 is a key regulator in the proliferation and differentiation of neural stem/progenitor cells (NSPCs) [17,18,19,20,21] as well as in the inflammatory response [22,23,24]. In stroke patients as well as rodent stroke models, miR-137 is highly downregulated and accompanied by a hyper-inflammatory response immediately after injury [9,22,25,26,27]. Moreover, miR-137 has been identified as a reliable biomarker of TBI [28,29]. However, whether miR-137 is a therapeutic target for TBI remains to be investigated.

Here, we report that early-stage application of exogenous miR-137 agomir (Agomir-137) dramatically repress gliogenesis and inflammatory responses, resulting in an improvement in neuronal survivability as well as motor recovery in mice with a motor cortex stab injury.

## 2. Results

### 2.1. Temporal Expression and Exogenous Intervention of miR-137 Following M1 Lesion

**Figure 1:**
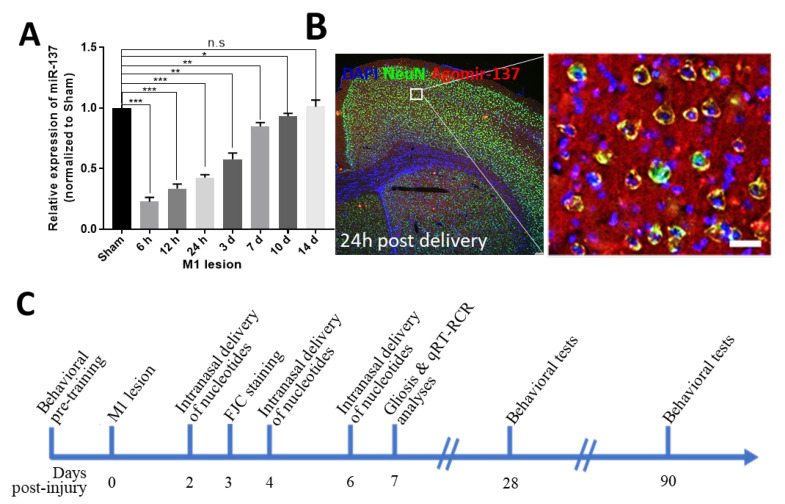
Temporal expression and intranasal administration of Agomir-137 following the M1 lesion. (**A**) Temporal changes in miR-137 expression level post M1 lesion according to RT-PCR. The expression of miR-137 was dramatically decreased in the early stage of M1-NSI. Starting from 7dpi. The expression level of miR-137 gradually recovered to a similar level to that in the sham control group at 14 dpi. Data are presented as means ± SEM. n = 4. * *p* < 0.05, ** *p* < 0.01, *** *p* < 0.001. n.s., non-significant. (**B**) Representative images of cerebral cortex from CY3-labeled Agomir-137-treated mice on day 1 after intranasal delivery. The region within the white box (left panel) is shown in a higher-magnification view in the right panel. Nuclei (DAPI^+^) and neurons (NeuN^+^) are stained blue and green, respectively. (**C**) Experimental scheme of miR-137 intervention and functional assays. After all mice received pre-training in behavioral tests, animals were then randomly divided into sham and M1 lesion groups. Agomir-137, Antagomir-137 or scramble nucleotides was administrated through the nasal route at 2, 4 and 6 dpi. FJC staining of brain sections was performed at 3 dpi. Gliosis and qRT-PCR analyses were conducted using brain tissues at 7 dpi. Finally, mice were subjected to a battery of behavioral tests at 28 and 90 dpi.

To examine any changes in miR-137 expression levels in the motor cortex following TBI, we chose 8-week-old male mice and performed needlestick injuries (NSIs) in the left primary cortex (M1). qRT-PCR analysis demonstrated that the expression of mature miR-137 was significantly downregulated in the motor cortex in the early stage (6 h to 7 d) of the M1 lesion (Figure 1A). At 14 days post injury (dpi), the miR-137 expression level had recovered to the same level as that in the sham group (Figure 1A). Since miR-137 loss of function is a well-known mechanism underlying the onset of various neurological diseases and intranasal administration of 1 nmol CY3-labeled Agomir-137 can be efficiently delivered to neurons as well as other cells in the brain (Figure 1B), we speculate that early-stage supplementation of miR-137 mimics might be a useful approach to protect against M1 lesions. 

### 2.2. Early-Stage Delivery of Agomir-137 Improves Locomotor Function after M1 Lesion

**Figure 2:**
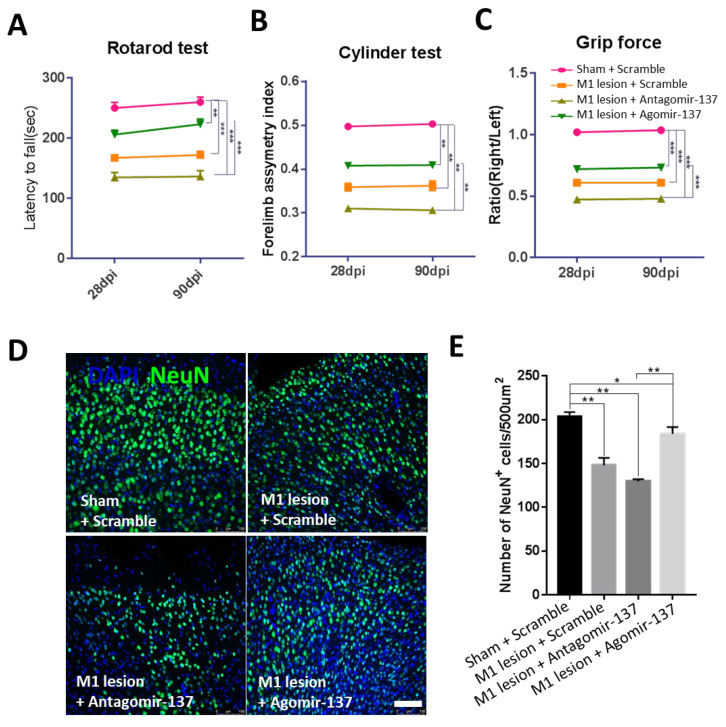
Early-stage (0 to 7 dpi) treatment of Agomir-137 promoted locomotor recovery and neuronal survival at 28 and 90 dpi. (**A**) Rotarod test showed that the mean time spent on accelerated rotarod in the M1 lesion + Agomir-137 group was significantly longer than that in the M1 lesion + Antagomir-137 and M1 lesion + Scramble groups at 28 and 90 dpi. n = 7 mice in the M1 lesion + Antagomir-miR-137 group; n = 8 mice in other groups. (**B**) Cylinder test showed that mice in the M1 lesion + Agomir-137 group preferred to use the right (affected) forelimb when compared to mice in the M1 lesion + Antagomir-137 or the M1 lesion + Scramble group at 28 and 90 dpi. n = 7 mice in the M1 lesion + Antagomir-miR-137 group; n = 8 mice in every other group. (**C**) Grip strength of the right (affected) forepaw showed a better recovery in the M1 lesion + Agomir-137 group than that in the M1 lesion + Antagomir-137 or the M1 lesion + Scramble groups at 28 dpi and 90 dpi. n = 7 mice in the M1 lesion + Antagomir-miR-137 group; n = 8 mice in every other group. (**D**,**E**) Representative images (**D**) and quantification of NeuN immunostaining (**E**) showed that neuronal density was significantly enhanced in the M1 lesion + Agomir-137 group compared to the M1 lesion + Scramble group at 90 dpi. n = 16 brain sections from 4 mice per group. Scale bar, 100 µm. Data are presented as means ± SEM. * *p* < 0.05, ** *p* < 0.01, *** *p* < 0.001.

Next, we tested whether exogenous intervention of miR-137 has an effect on mice with M1 lesion. We intranasally delivered Agomir-137, Antagomir-137 or scramble molecules via the nose-to-brain route to mice every other day at the early stage of M1 lesion (0–7 dpi) and performed behavioral analysis as well as immunostaining at 28 and/or 90 dpi (Figure 1C). The rotarod test, cylinder test and forepaw grip force test indicated that early-stage delivery of Agomir-137 enhanced locomotor recovery, while early-stage delivery of Antagomir-137 deteriorated locomotor impairments at 28 and 90 dpi (Figure 2A–C). Consistently, early-stage supplementation of Agomir-137 increased but Antagomir-137 decreased the number of neurons in the M1 region at 90 dpi (Figure 2D,E). These results demonstrate that intranasal delivery of Agomir-137 at the early stage has effective therapeutic potential for M1 lesions. 

### 2.3. Early-Stage Delivery of Agomir-137 Reduces Neural Apoptosis and Gliosis following M1 Lesion

**Figure 3:**
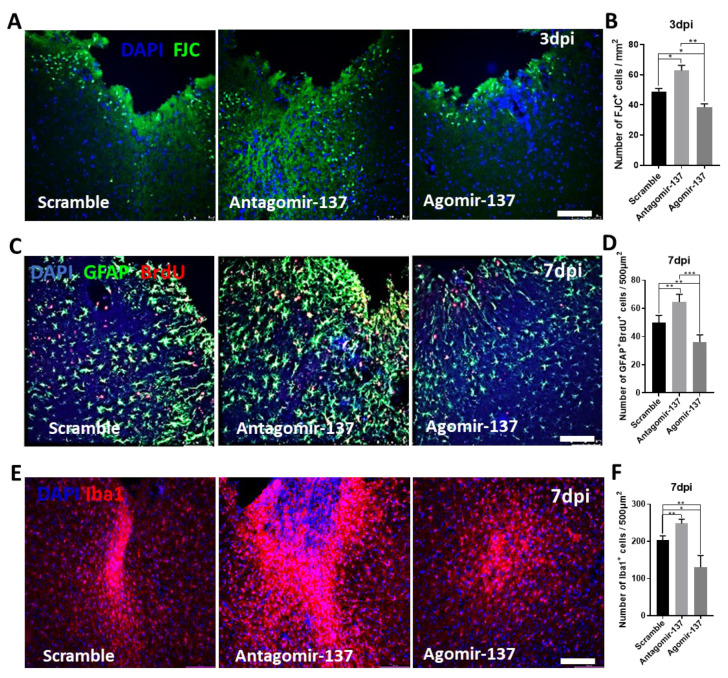
Intranasal delivery of Agomir-137 inhibited neuronal apoptosis and gliosis in the early stage of M1 lesion. (**A**,**B**) Representative images (**A**) and quantification (**B**) of FJC staining indicated that Agomir-137 inhibited neuronal apoptosis in M1 at 3 dpi. (**C**,**D**) Representative images (**C**) and quantification (**D**) of GFAP and BrdU immunostainings demonstrated that astrogliosis was decreased at 7 dpi in the M1 lesion + Agomir-137 group. (**E**,**F**) Representative images (**E**) and quantification of Iba1 immunostaining (**F**) indicated that the number of microglia/macrophages was dramatically reduced at 7 dpi in M1 by Agomir-137 administration. Scale bars, 100 µm. Data are presented as means ± SEM. n = 16 brain sections from 4 mice per group. * *p* < 0.05, ** *p* < 0.01, *** *p* < 0.001.

To further explore the roles and mechanisms of Agomir-137 in locomotor recovery and neuronal survival, we performed FJC, GFAP and Iba1 immunostainings of M1 tissues to determine whether Agomir-137 has an effect on neuronal apoptosis and hyperactive gliosis in the early stage of an M1 lesion. As we expected, the number of FJC^+^ cells was significantly decreased in the M1 lesion + Agomir-137 group, while the number of FJC^+^ cells was dramatically increased in the M1 lesion + Antagomir-137 group compared to that in the M1 lesion + Scramble group (Figure 3A,B), showing that Agomir-137 inhibited neuronal apoptosis in early stage post TBI. Meanwhile, Agomir-137 treatment reduced the number of both astrocytes (Figure 3C,D) and microglia/macrophages (Figure 3E,F) in the M1 region at 7dpi, while gliosis was exacerbated in the M1 lesion + Antagomir-137 group, indicating that Agomir-137 treatment repressed hyper-activated gliosis. 

### 2.4. Early-Stage Delivery of Agomir-137 Reduces the Expression of Pro-Inflammatory Genes

**Figure 4:**
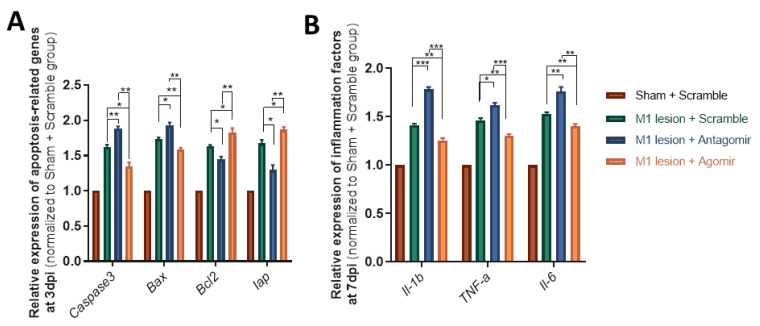
Early-stage delivery of Agomir-137 repressed the expression of pro-apoptotic and pro-inflammatory genes following M1 lesion. (**A**) qRT-PCR analysis showed decreased expression of pro-apoptotic genes (*Caspase3* and *Bax*) and increased expression of anti-apoptotic genes (*Bcl2* and *Iap*) in M1 tissues in the M1 lesion + Agomir-137 group at 3 dpi. (**B**) qRT-PCR analysis indicated that the expression of pro-inflammation factors was significantly decreased in M1 tissues in the M1 lesion + Agomir-137 group at 7 dpi. Data are presented as means ± SEM. n = 4 mice per group, * *p* < 0.05, ** *p* < 0.01, *** *p* < 0.001.

At the molecular level, we observed that *Caspase3* and *Bax* decreased, but *Bcl2* and *Iap* (anti-apoptotic genes) had increased expression in M1 tissues in the M1 lesion + Agomir-137 group compared to the M1 lesion + Scramble group at 3 dpi (Figure 4A). Meanwhile, the mRNA expression levels of pro-inflammatory factors (such as IL-1β, IL-6 and TNF-α) were significantly repressed in M1 tissues in the M1 lesion + Agomir-137 group compared to that in the M1 lesion + Scramble group at 3dpi (Figure 4B). On the contrary, Antagomir-137 treatment resulted in downregulation of *Bcl2* and *Iap* (Figure 4A), as well as the upregulation of *Caspase3*, *Bax*, IL-1β, IL-6 and TNF-α (Figure 4A,B) compared to the M1 lesion + Scramble group at 3dpi. These data suggested that early-stage delivery of Agomir-137 inhibited neuronal apoptosis and gliosis through the repression of pathways of pro-apoptosis and pro-inflammation after an M1 lesion.

## 3. Materials and Methods

### 3.1. Mice

C57BL/6J mice were obtained from the SPF (Beijing, China) Biotechnology Company and maintained with a 12 h light/dark cycle. All animal procedures were approved by the Animal Committee of the Institute of Zoology, Chinese Academy of Sciences (Approval Code: 2020-008; Approval Date: 28 March 2020).

### 3.2. Motor Cortex Stab Injury

Thirty-two mice (8 weeks old) in total were randomly divided into 4 groups (n = 8 per group), namely Sham + Scramble, M1 lesion + Scramble, M1 lesion + Antagomir-137 and M1 lesion + Agomir-137 groups. A primary motor cortex stab injury (M1 lesion) was performed as previously described [30]. Briefly, 8-week-old male mice were anesthetized and received needlestick injuries in the left primary cortex (M1) in a KOPF stereotaxic apparatus. Three evenly spaced holes (0.9 mm in diameter) were drilled in the skull just above the left M1 region to produce a traumatic lesion. Each hole was subjected to two needlestick wounds with a 26 G syringe needle inserted 1.5 mm deep. The holes were cleaned with a disposable alcohol swab and sealed with bone wax. For the sham treatment, the same surgical procedures were performed only without a needlestick injury. 

### 3.3. Intranasal Delivery of Agomir-137/Antagomir-137

Immediately after TBI, intranasal administration of Agomir-137, Antagomir-137 or scramble (negative control) nucleotides (RIBO Biotech, Guangzhou, China) [31,32,33] was performed. The nucleotides were dissolved in 24 µL of RNase-free water to reach a final concentration of 1 nM. The working drug solution was administered by pipette in 4 µL drops (total of six fractions), alternating between each nostril every 2–3 min. Control mice received equal dosages of scramble nucleotides (negative control). The intranasal delivery was performed every other day for a total of 7 days post M1 lesion.

### 3.4. qRT-PCR

TRIzol reagent (Invitrogen, Carlsbad, CA, USA) was used for total RNA isolation. The RNA quality of all samples met a 260/280 nm ratio >2.0 and 260/230 nm ratio in the range of 2.0–2.2, as was assessed with the Thermo NanoDrop 2000 spectrophotometer. cDNA reverse transcription (2 µg total RNA per sample) was performed using the Transcriptor First Strand cDNA Synthesis Kit (TransGen Biotech, Beijing, China). cDNA was quantified using the SYBR Green assay, and the relative gene expression levels were calculated by using the ∆∆Ct method. GAPDH was used as the internal control. The primers we used for qRT-PCR are listed in Table 1.

### 3.5. Immunostaining

Mice perfusion was performed after anaesthetization. Cold-phosphate-buffered saline (PBS) and 4% paraformaldehyde (PFA) in PBS (pH 7.4) were used for blood clearance and tissue fixation, respectively. Brains were soaked in 4% PFA overnight for post-fixation and in 30% sucrose for equilibration. Brain sections (40 µm thick) were washed in PBS and then soaked in blockage reagent (3% BSA, 0.3% Triton X-100 and 0.2% sodium in PBS) for 1h at room temperature. Following incubation overnight with primary antibodies at 4 °C, brain sections were washed with PBS and then incubated with the secondary antibodies conjugated with Alexa Fluor 488 or 594 (1:500). The primary antibodies we used were as follows: anti-GFAP (Proteintech, Rosemont, IL, USA; 16825-1-AP; 1:1000); anti-Iba1 (Wako, Richmond, VA, USA; #019-19741; 1:1000); and anti-NeuN (Millipore, Hongkong, China; ABN78; 1:1000). Finally, sections were mounted on glass slides with antifade mounting medium. Confocal images were captured using a ZEISS 710 confocal laser-scanning microscope. Image analyses were performed using ImageJ software v1.54d (NIH, Bethesda, MD, USA).

### 3.6. Fluoro-Jade C Staining

Brains were sectioned into 30 µm thick portions for Fluoro-Jade C (FJC, a sensitive and specific fluorescent marker of neuronal degeneration) staining. Brain sections were rinsed in 80% ethanol containing 1% sodium hydroxide, 70% ethanol and distilled water for penetration. Then, brain sections were incubated in 0.1% FJC solution in the dark for 20 min and mounted in mounting medium.

### 3.7. Behavioral Tests

Behavioral analysis was performed as previously described [30]. One mouse in the M1 lesion + Antagomir-miR-137 group died before the start of behavioral tests. All the other mice were transported to the behavioral facility 1d before the assays for acclimation. All experimental instruments were cleaned using 70% ethanol between mice to avoid odor disturbance. Behavioral videos were recorded and analyzed using the software Smart V3.0.03 (Panlab, Barcelona, Spain).

Rotarod test. At the beginning, every mouse was allowed to remain stationary on the spindle for 10 s at 0 rpm. The rotational speed was then slowly accelerated to 40 rpms within 30s. The mouse remained on the spindle at 40 rpms until the 5 min test period elapsed. Each mouse went through four trials with at least 30 min between trials.

Cylinder test. Every mouse was gently placed into a non-reflective plexiglass cylinder (15 cm in diameter, 35 cm high), and the number of paw-contacts with the cylinder wall within 5 min was recorded with a digital camera. Every time a paw touched the cylinder wall was recorded. The percentage of contralateral wall touches among total contralateral and ipsilateral touches was calculated. 

Forepaw grip strength test. Animals usually have progressive atrophy of the motor cortices and degeneration of the corticospinal tracts after TBI [34]. Poor grip strength is a cardinal feature of various animal models with an M1 injury [30,35,36]. To measure the grip strength of a forepaw, the opposite forepaw was covered with an adhesive tape (2 × 2 cm), and the mouse was held parallel to the bar of the grip strength meter. Once the unrestrained forepaw reliably grasped the bar, the mouse was then gently pulled away from the device by the base of the tail. The grip strength was measured five times for each forepaw with an inter-trial rest period of 15 s and determined according to the mean maximum force generated in five trials. The impairment of muscle force of an animal was evaluated by the ratio of right/left forepaw grip strength. 

### 3.8. Statistical Analysis

All statistical analyses were conducted using the software GraphPad Prism v7.2 (San Diego, CA, USA). Datasets were analyzed for significance using a One-Way ANOVA with Dunnett’s multiple comparisons test. Sample sizes are provided in the figure legends. All data are presented as mean ± SEM. When the *p*-value is less than 0.05, the results are determined as statistically significant. 

## 4. Discussion

TBI is a complex pathogenesis process which is caused by primary and secondary injuries [37]. The primary injury happens immediately post TBI and causes tissue damage, which is accompanied by neuronal cell death and hyper-activated gliosis. Neuroinflammation can cause an acute secondary injury phase that lasts days to months after the primary injury [38,39]. Unfortunately, an anti-inflammatory agent which can improve TBI outcomes in clinical trials is still lacking [40]. The present study elucidated the therapeutic potential of Agomir-137 in the resolution of neuroinflammation and locomotor recovery after motor cortex stab injury.

TBI induces increased levels of pro-inflammatory cytokines such as TNF-α, IL-1β and IL-6 [41,42,43] in the early stage of injury, causing progressive neurodegeneration [44]. Several studies have shown that miR-137 is an inflammation repressor under various disease conditions. During rheumatoid arthritis (RA) pathogenesis, miR-137 is downregulated in association with the REST/mTOR axis, which is negatively correlated with inflammatory factors [45]. In rat cartilage tissue of osteoarthritis, miR-137 is also downregulated, and its over-expression can reduce the inflammatory factors (TNF-α, IL-1β, IL-6) via downregulating the TCF4-AMPK/NF-κB pathway [46]. In the mouse model of spinal cord injury (SCI), CircRNA3616 upregulates TLR4 expression by sponging miR-137, and CircRNA3616 knockdown attenuates neuroinflammation via the TLR4/NF-κB pathway after SCI [47]. Moreover, Gao and colleagues reported that miR-137 is downregulated and its downstream target NeuroD1 is upregulated in mouse microglial BV2 cells under LPS challenge, resulting in a higher expression of TNF-α, IL-1β and iNOS [48]. Consistent with this, our study shows that early-stage delivery of Agomir-137 can effectively repress the expression of pro-inflammatory factors, but the underlying molecular mechanisms remain to be investigated in the future. 

Focal brain lesions induce glial scar formation and neuronal death, and they are present in 20~25% of all people who incur a TBI [49]. Immediately after injury, astrocytes and microglia proliferate and intertwine to form a glial scar that separates from healthy tissue [50,51]. Although the glial scar serves as a physical barrier to limit the detrimental effects of fibrotic tissue and macrophages after the acute stage of brain injury, several studies have suggested that a moderate reduction in the density without interrupting the integrity of the glial scar improves axonal regeneration and functional recovery [52,53]. It seems that our present study supports this hypothesis. Early-stage delivery of Agomir-137 reduces the cell densities of both microglia/macrophages and astrocytes, while motor function is significantly improved in Agomir-137-treated mice after an M1 lesion. miR-137 is a well-known suppressor of cell proliferation, mainly by regulating the cell cycle and enhancing differentiation of glioma stem cells via its downstream targets, such as *Clcl12*, *Cox2*, *Pdgfr*, *Sp1*, *Tcf4* and/or *Cdk6* [54]. We speculate that Agomir-137 may directly inhibit the proliferation of glial cells following M1 injuries, but future studies are needed to test this hypothesis and to identify the downstream targets that are responsible for regulating glial scar formation. 

It is worth noting that this study has several limitations. Firstly, despite of the important roles of miR-137 in neurogenesis, synaptic transmission and plasticity have been experimentally elucidated under health and disease conditions [48,55,56,57]; thus, it would be interesting to examine whether there is a potential benefit of Agomir-137 administration in enhancing neurogenesis and synaptic plasticity after an M1 lesion. Secondly, since the intranasal route not only transports drugs directly to the brain from the nasal cavity along the olfactory and trigeminal nerves [58] but also delivers drugs to the blood circulation [59], future works are needed to evaluate the systemic and CNS side-effects of Agomir-137. Finally, as our M1 lesion model belongs to focal type of injury, additional experiments are required to ascertain the therapeutic potential of Agomir-137 in the diffuse type of TBI.

In summary, our findings, for the first time, implicate the therapeutic potential of Agomir-137 in motor functional recovery after TBI. Given that miR-137 loss of function is responsible for the pathogeneses of several neurodevelopmental and neuropsychiatric disorders [60,61], it would be interesting to examine whether Agomir-137 administration is also beneficial for the treatment of these devastating diseases. 

## Figures and Tables

**Table 1:** Primers used for qRT-PCR.

Gene		Primer Sequence (5′-3′)
*miR-137*	Forward	CGCGCGTTATTGCTTAAGAATAC
	Reverse	AGTGCAGGGTCCGAGGTATT
*Actin*	Forward	TGCACCACCAACTGCTTAG
	Reverse	GGATGCAGGGATGATGTTC
*TNF-α*	Forward	ACGGCATGGATCTCAAAGAC
	Reverse	GTGGGTGAGGAGCACGTAGT
*IL-1β*	Forward	CAGGCAGGCAGTATCACTCA
	Reverse	TGTCCTCATCCTGGAAGGTC
*Caspase-3*	Forward	TGGTGATGAAGGGGTCATTTATG
	Reverse	TTCGGCTTTCCAGTCAGACTC
*Bcl2*	Forward	GTCGCTACCGTCGTGACTTC
	Reverse	CAGACATGCACCTACCCAGC
*Bax*	Forward	TGAAGACAGGGGCCTTTTTG
	Reverse	AATTCGCCGGAGACACTCG
*IL-6*	Forward	ATGGATGCTACCAAACTGGAT
	Reverse	TGAAGGACTCTGGCTTTGTCT
*Iap*	Forward	TGGGCACAGCTTATCTGGC
	Reverse	TGACTATGGTCAGAGTGTCGC

## Data Availability

All datasets supporting the conclusions are included in the article. Further enquiries on data and materials can be directed to the corresponding author.
